# Frequency-multiplexed optical reservoir computing using a microcomb

**DOI:** 10.1515/nanoph-2025-0260

**Published:** 2025-08-29

**Authors:** Jonathan Cuevas, Yue Hu, Baoqi Shi, Junqiu Liu, Kaoru Minoshima, Naoya Kuse

**Affiliations:** Graduate School of Sciences and Technology for Innovation, Tokushima, Japan; International Quantum Academy, Shenzhen, China; Hefei National Laboratory, University of Science and Technology of China, Hefei, China; Graduate School of Informatics and Engineering, The University of Electro-Communications, Chofu, Japan; 13109 Institute of Post-LED Photonics, Tokushima University, Tokushima, Japan; 13109 Institute of Photonics and Human Health Frontier, Tokushima University, Tokushima, Japan

**Keywords:** optical frequency comb, reservoir computing, microresonator

## Abstract

Optical reservoir computing (ORC) promises fast, energy-efficient temporal inference by harnessing the rich transient dynamics of photonic systems. Yet most ORC demonstrations still depend on fiber delay lines or camera-based spatial multiplexing, which caps the clock rate at a few tens of MSa/s and complicates monolithic integration. Here we introduce a frequency-multiplexed ORC whose nodes are the individual modes of a dissipative Kerr-soliton microcomb generated in a high-*Q* Si_3_N_4_ microresonator. The input signal is encoded as a rapid detuning modulation of the pump laser, so the intracavity dynamics of the microcomb provide both the high-dimensional nonlinear mapping and tens of nanoseconds of memory, while output weighting is realized optically with standard microring arrays. Numerical modeling with 60 comb modes provides a normalized mean-square error (NMSE) of 0.015 on the Santa Fe chaotic time-series task at 50 MSa/s and more than a tenfold reduction in symbol-error rate for nonlinear equalization (NLEQ) at 100 MSa/s. A proof-of-concept experiment using 37 measured modes also confirms the concept on the Santa Fe chaotic time-series and NLEQ benchmarks. Because both the microcomb and weighting network are fabricated by a complementary metal-oxide semiconductor (CMOS)-compatible process, the architecture offers a clear path toward compact, energy-efficient photonic processors operating at greater than 1 GSa/s, directly addressing the scalability and speed challenges of nanophotonic ORC.

## 1 Introduction

Reservoir computing (RC) has emerged as a powerful computational framework that harnesses the rich, nonlinear dynamics of high-dimensional systems to tackle complex problems with minimal training effort. First introduced for recurrent neural networks [1], RC projects input signals into a high-dimensional reservoir whose transient responses can be exploited for pattern recognition, time-series prediction, classification, and related tasks. A key attraction of RC is its training efficiency: only the output weights are adjusted, while the internal reservoir dynamics remain fixed, providing a highly practical route toward hardware implementation.

Building on this concept, optical reservoir computing (ORC) has garnered considerable interest thanks to its intrinsic advantages in speed, massive parallelism, and energy efficiency [2]. Leveraging the ultrafast dynamics and broad bandwidth of photonic systems, ORC provides a compelling platform for real-time processing of high-dimensional data streams, which is particularly valuable for optical communications, real-time signal processing, and adaptive control. In ORC architectures, different degrees of freedom of light are employed as the reservoir nodes. The first ORC demonstrations adopted time-multiplexed schemes [3], [4], [5], [6]. These approaches create a high-dimensional reservoir using only minimal hardware by using a single nonlinear node combined with delayed feedback. Typically, an input-modulated laser feeds an optical-fiber delay line arranged in a loop that is closed either through an opto-electro-optic conversion stage [3], [4] or by reinjection into the laser cavity [5], [6]. The fiber delay line imposes temporal separation between successive virtual nodes, transforming the input into a high-dimensional representation. The delayed feedback provided by the loop, together with the nonlinearity of the laser or the opto-electro-optic conversion, supplies both the memory and nonlinear processing required for RC. While time-multiplexing has been proven effective, its scalability and processing speed are ultimately constrained by the fiber-based architecture – for example, a 10-m delay line limits the input modulation rate to roughly 20 MSa/s. These limitations force a trade-off between the number of accessible virtual nodes and the system’s attainable modulation rate. In contrast, spatial-multiplexed ORC realizes the reservoir with an array of discrete physical nodes distributed across space [7], [8], [9], [10], [11]. A laser that is modulated either spatially or temporally is projected into a high-dimensional state by, for example, diffractive optical elements [7], multimode fibers or waveguides [8], [9], [10], or coupled microring resonators [11]. Recent demonstrations have simplified the core components, paving the way for scalable implementations based on integrated-photonics platforms [9], [12]. However, embedding memory into spatial-multiplexed reservoirs is non-trivial: the reliance on cameras and spatial light modulators [7], or on supplementary time-multiplexing combined with digital signal processing [9], caps the clock speed and therefore the overall throughput. Chip-scale optical RC using spiral waveguides has also been demonstrated [13]. Although the RC can be complementary metal-oxide semiconductor (CMOS)-compatible, the number of effective nodes is ultimately limited by the footprint of the spiral waveguides. In addition, because the output nodes are physically separated, most spatially multiplexed schemes rely on two-dimensional cameras or on photodetector and analog-to-digital converter arrays. These devices limit the modulation rate, which can fall to only a few kSa/s when cameras are used, and they also make the hardware architecture more complex.

A third degree of freedom for ORC is frequency. Frequency-multiplexed schemes exploit the spectral domain, using the individual frequency components of light as virtual nodes [14], [15]. In these demonstrations, an electro-optic frequency comb (EO comb) supplies a set of uniformly spaced spectral lines, each serving as a distinct node. The nonlinear interactions essential to RC are produced by optical phase modulation or cavity-based nonlinearities, which simultaneously furnish the short-term memory. Reservoir evolution therefore takes place entirely in the frequency domain, with the internal dynamics set by spectral mixing among the comb modes driven by an electro-optic modulator. Read-out is accomplished by passing the comb through a programmable spectral filter and detecting the selected components with a single photodiode, so the output weights are realized optically via controlled attenuation. Compared with time- and spatial-multiplexed architectures, this approach dispenses with an input mask and avoids photodiode/analog-to-digital converter (ADC) arrays, enabling higher potential clock rates and simpler hardware. Nevertheless, the modulation speed is still bounded by the round-trip time of the fiber cavity (≈20 MSa/s), and full integration on a photonic chip remains technically challenging, limiting current scalability.

In this work, we propose and validate a frequency-multiplexed ORC that uses a microcomb generated in a high-*Q* microresonator. This approach points toward fully integrated chip-scale ORC implementations. The pump CW laser, carrying the input waveform, excites the microresonator and is converted into a broadband microcomb. The resulting comb modes form a high-dimensional space, and photon storage in the high-*Q* cavity provides the short-term memory. Both the microcomb and the optical weighting network can be fabricated with CMOS-compatible processes [16], [17], [18], which offer a straightforward path to large-scale integration. Through numerical simulation and experiment, we show that the proposed architecture performs on the Santa Fe chaotic time-series prediction benchmark and on a nonlinear equalization (NLEQ) task, confirming its effectiveness and versatility.

## 2 Working principle

**Figure 1:**
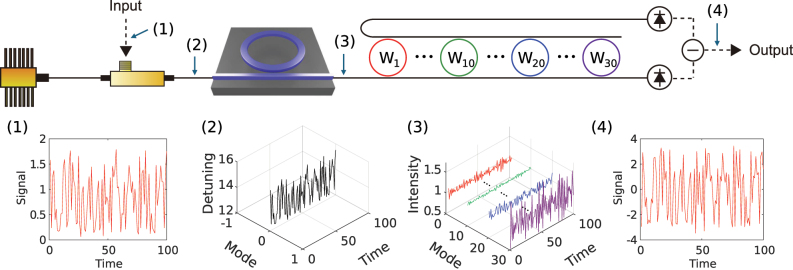
Conceptual illustration of the proposed ORC. Input signals (1) are imposed on the frequency of a pump continuous wave (CW) laser through an electro-optic modulator (EOM), thereby modulating the frequency detuning between the pump CW laser and a resonance of a microresonator (2). A power modulated soliton comb (3) is generated from a microresonator with the modulated pump CW laser. Optimized output weights (*W*_*i*_: *i* is the comb mode number) with both positive and negative values are applied via the use of drop and through ports of microring arrays, whose resonances are allocated for each comb mode. With the summation of the temporal waveforms of the weighted comb modes by a balanced photodetector, an output signal (4) is obtained.

Figure 1 illustrates the basic concept of our ORC. We use a dissipative Kerr soliton comb, not a chaotic comb, operating in a mode-locked state to ensure consistency, meaning that identical input signals produce identical outputs [19], [20]. A soliton comb is generated by coupling a single-frequency CW laser into a high-*Q* microresonator. After a single-soliton comb is deterministically established, the frequency of the pump CW laser is modulated by an electro-optic modulator driven by the input signal. The elements of the input sequence are time aligned, as indicated in panel (1) of Figure 1. This frequency modulation changes the detuning between the pump laser and the resonance of the microresonator, as illustrated in panel (2) of Figure 1. In this depiction, mode 0 denotes the pump mode. Modulating the detuning modifies the optical spectrum of the soliton comb [21] and produces comb-mode-dependent intensity modulation, as shown in panel (3) of Figure 1. The one-dimensional input signal is expanded into a higher-dimensional space equal to the number of comb modes through this process. Photon storage in the microresonator supplies short-term memory provided that $\frac{1}{f_{m}}$ , where *f*_*m*_ is the modulation rate, is shorter than the photon lifetime in the cavity [22]. In other words, *f*_*m*_ must exceed the resonance linewidth of the microresonator. However, if *f*_*m*_ is chosen excessively large, the resulting memory time becomes too long, which can degrade the performance of the ORC. The intensities of the comb modes are then weighted by an array of microrings whose resonances are aligned with the individual comb modes. These weights are optimized during training. Add-drop ports followed by a balanced photodiode allow the use of both positive and negative weights [23]. Finally, the system output is taken from the balanced photodiode, as illustrated in panel (4) of Figure 1.

## 3 Results

### 3.1 Numerical demonstration

Numerical simulations are conducted using the Lugiato–Lefever equation [24], [25]:

(1) $$\frac{\partial }{\partial t}\psi =-\left(1+i\delta \right)\psi +i|\psi {|}^{2}\psi +i\frac{D_{2}}{2!}{\left(i\frac{\partial }{\partial \theta }\right)}^{2}\psi +F.$$

Here *ψ* denotes the intracavity electric field. The variables *t* and *θ* represent slow time and fast time. Slow time describes how the field evolves over successive round trips inside the resonator, whereas fast time labels the angular position inside the cavity in a co-moving reference frame. The parameters *δ*, *D*_2_, and *F* correspond to the normalized detuning, normalized second-order dispersion, and normalized pump amplitude. The normalized pump power (*F* ^2^) and second-order dispersion are fixed at 32 and 0.048, values that match the experimental microresonator. On the physical time scale, we assume a free spectral range of 100 GHz and a loaded resonance linewidth of 28 MHz, again consistent with the device used in the experiments. When input signals are applied, *δ* becomes a function of the slow time.

**Figure 2:**
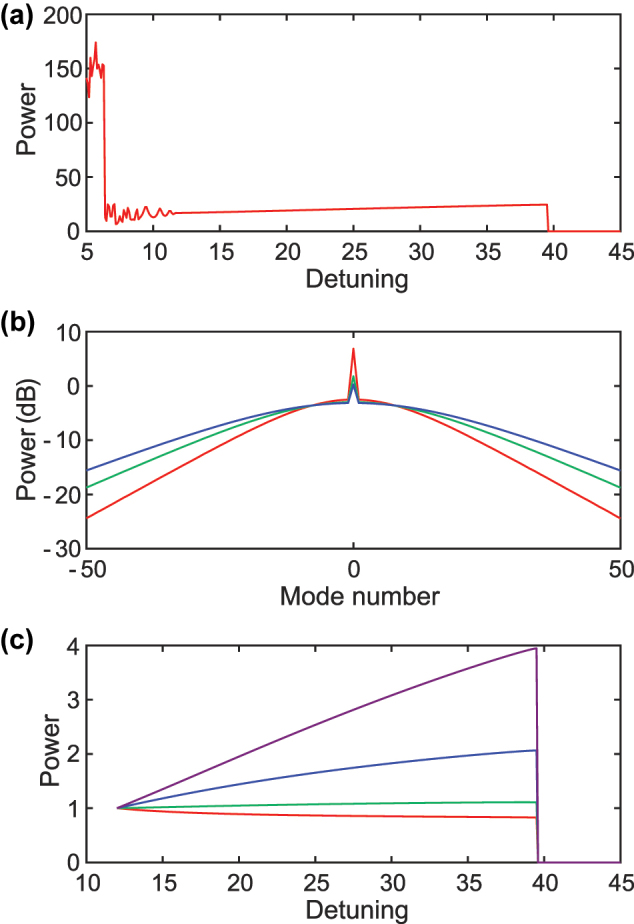
Static response of the simulated soliton microcomb to pump-laser detuning. (a) Total comb power versus detuning. (b) Optical spectra of the soliton comb at the detuning of 13 (red), 23 (green), and 33 (blue). (c) Normalized powers of selected comb modes as a function of detuning. The comb mode numbers are +1st (red), +10th (green), +20th (blue), and +30th (purple).

**Figure 3:**
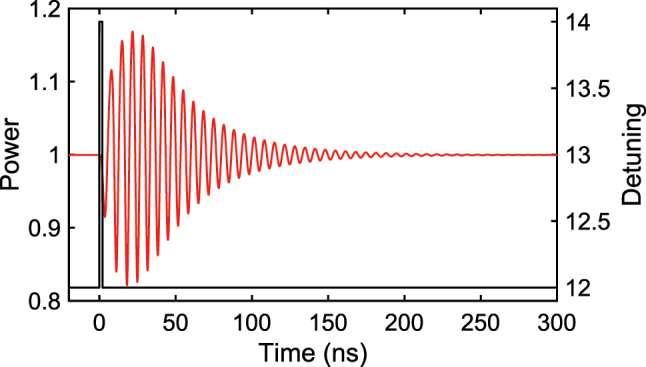
Transient response of the power of the −1st comb mode (red) when the detuning is stepped from 12 to 14 for 2 ns (black).

First, we examine the static behavior of a soliton comb while varying the detuning. With the fixed pump power *F* ^2^ = 32 a soliton exists for detuning values between 12 and 39, as illustrated in Figure 2(a). Figure 2(b) shows the optical spectra obtained at detunings of 13 (red), 23 (green), and 33 (blue). These spectra are symmetric because higher-order dispersion and the Raman effect are omitted in the simulation. For positive comb mode numbers, we observe that the power of comb modes below 7 decreases as the detuning increases, whereas the power of comb modes above 7 grows when the detuning is reduced. Figure 2(c) plots the normalized power of the +1st (red), +10th (green), +20th (blue), and +30th (purple) modes versus detuning, scaled to one at the initial detuning of 12. The power change is more pronounced when the detuning is smaller (that is, when the pump frequency lies closer to the cavity resonance). The distinct and nonlinear power responses of the different modes indicate rich dynamics that are promising for RC. We next analyze the dynamic response. The detuning is abruptly stepped from 12 to 14 for 2 ns, a duration shorter than the photon lifetime $\left(\approx \frac{1}{linewidth}\right)$ . Figure 3 reveals that the power of the −1st comb mode continues to evolve for more than 100 ns and exhibits oscillations with a period of 6.8 ns. The prolonged response results from photon storage in the microresonator, and the oscillatory behavior arises from the interplay between the Kerr nonlinearity and the changing intracavity power.

**Figure 4:**
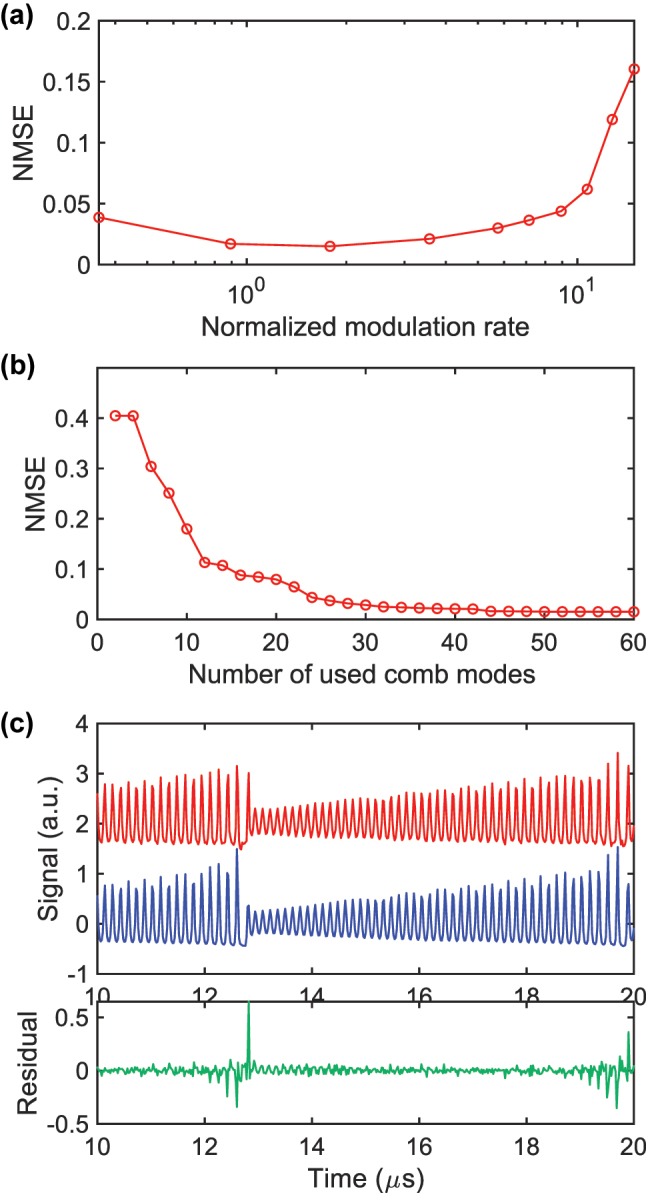
Optimization of NMSE for numerical Santa Fe prediction. (a) NMSE when modulation rate is varied for *N*_comb_ = 60. Normalized modulation rate is defined as $\frac{f_{m}}{linewidth}$ . (b) NMSE when *N*_comb_ is varied with the normalized modulation rate fixed at ≈2. (c) Predicted (red) and target (blue) Santa Fe time-series waveforms, and the residual (green) between them, when *N*_comb_ and normalized modulation rate are 60 and around 2, respectively.

As a first benchmark we employ the one-step-ahead Santa Fe time-series prediction task. This chaotic benchmark was introduced during the Santa Fe Institute competition, where the data come from intensity fluctuations of a far-infrared NH_3_ laser. After a soliton comb is generated at a detuning of 12, the detuning is swept between 12 and 18 in response to the input signal. The detection sampling rate is 5 GSa/s. Up to 60 comb modes are used, and the intensity of each mode is normalized to one in the absence of modulation. Only one side of the comb is taken because the opposite side shows the same response when the Raman effect and third-order dispersion are neglected. This is not true for the experiment, and both sides of the comb modes are used in the experiment. The reservoir outputs, that is the intensities of the individual comb modes, are trained on 3,000 samples with ridge regression to obtain the optimal weights, and a further 1,000 samples are used for test. To determine the best modulation rate, we evaluate normalized mean-square errors (NMSEs) with *N*_comb_ = 60 while varying the modulation rate, as shown in Figure 4(a):

(2) $$NMSE=\frac{1}{N}\overset{N}{\underset{n=1}{\sum }}\frac{{\left(d\left(n\right)-y\left(n\right)\right)}^{2}}{\sigma^{2}\left(d\right)}.$$

Here *N* is the number of samples, and *d* and *y* are the target and predicted signals. *σ* ^2^(∗) denotes the variance of *. The minimum NMSE occurs when the normalized modulation rate $\frac{f_{m}}{linewidth}$ is about 2, corresponding to 50 MSa/s for a resonance linewidth of 28 MHz, which shows that the short-term memory is used effectively. NMSE increases gradually up to a normalized rate of 10 and then rises sharply for higher rates, behavior that matches earlier observations that excessive memory degrades performance on the Santa Fe task. Figure 4(b) plots NMSE as a function of *N*_comb_ with the normalized modulation rate fixed near 2. Increasing *N*_comb_ markedly improves performance, reaching an NMSE as low as 0.015. The best predicted Santa Fe waveform (red) is compared with the actual data (blue) in Figure 4(c). The prediction follows the target closely, and most residuals lie between −0.1 and +0.1.

**Figure 5:**
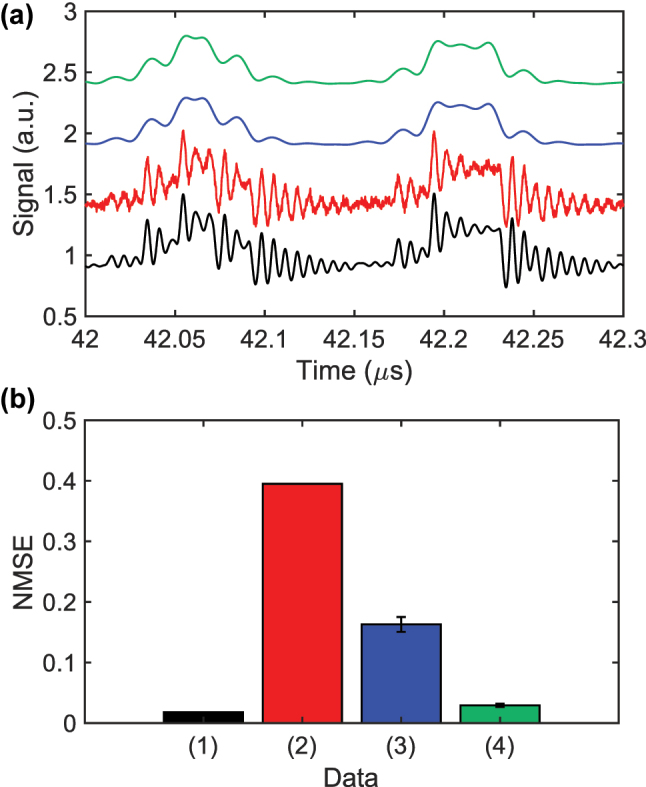
NMSE for numerical Santa Fe prediction under four post-processing schemes. (a) Normalized power of the −30th comb mode when the detuning is driven by the Santa Fe signal under four conditions: noise-free, no low-pass filter (LPF) (black); noisy, no LPF (red); noise-free, LPF applied (blue); noisy, LPF applied (green). (b) Corresponding NMSE values: (1) no noise/no delay/no LPF; (2) noise/no delay/no LPF; (3) noise/delay/no LPF; (4) noise/delay/LPF. *σ*_*G*_ = 0.02, *N*_comb_ = 60, and normalized modulation rate ≈2.

The results in Figure 4 are effectively noise-free except for a small amount of random noise added during each round trip. In a real soliton comb, however, amplified spontaneous emission (ASE) from the optical amplifiers placed before and after the microresonator raises the noise floor. To mimic these conditions, we add Gaussian noise with standard deviation *σ*_*G*_ = 0.02 to the comb-mode intensities, as shown by the red trace in Figure 5(a). This level corresponds to a signal-to-noise ratio (SNR) of 34 dB at 0.02 nm resolution bandwidth (RBW) in the telecom band. With this noise present the NMSE increases by about 0.4, as indicated by trace (2) in Figure 5(b). We explore two mitigation strategies. First, we insert random delays between comb modes, with the maximum delay set to $\frac{1}{2f_{m}}$ . These delays reduce the NMSE to 0.163, trace (3) in Figure 5(b), and the error bar gives the standard deviation across ten independent delay patterns. The random delays enhance the temporal complexity of the comb-mode power waveforms, similar to time-division multiplexing, without increasing the number of nodes. Second, we apply a low-pass filter (LPF) with a 75 MHz cutoff to the comb-mode powers. The filter attenuates the noise according to

(3) $$\sigma_{\text{LPF}}=\sigma_{\text{G}}\sqrt{\frac{2f_{\text{LPF}}}{f_{s}}}.$$

Here *σ*_LPF_ is the noise standard deviation after filtering, and *f*_LPF_ and *f*_*s*_ are the LPF cutoff frequency and the ADC sampling rate. Because of the LPF, the impact of noise is less pronounced, as indicated by the blue and green curves in Figure 5(a). Combining the random delays with LPF processing lowers the NMSE of the noise-corrupted comb-mode waveforms to 0.0293, as shown by trace (4) in Figure 5(b).

Next we apply our ORC to a nonlinear equalization (NLEQ) task. The NLEQ problem is common in digital communications, where the goal is to remove nonlinear distortion and inter-symbol interference produced by the channel and to recover the transmitted symbols. In the model the original bit sequence *d*(*i*), which takes the values −3, −1, +1, and +3, is mixed by inter-symbol interference according to

(4) $$\begin{array}{cc} q\left(i\right) & =0.08\;d\left(i+2\right)-0.12\;d\left(i+1\right)+d\left(i\right)\\ & \;+0.18\;d\left(i-1\right)-0.1\;d\left(i-2\right)+0.091\;d\left(i-3\right)\\ & \;-0.05\;d\left(i-4\right)+0.04\;d\left(i-5\right)\\ & \;+0.03\;d\left(i-6\right)+0.01\;d\left(i-7\right). \end{array}$$

The resulting sequence *q*(*i*) is then distorted nonlinearly and corrupted by Gaussian noise *v*(*i*), giving

(5) $$u\left(i\right)=q\left(i\right)+0.036\;q{\left(i\right)}^{2}-0.011\;q{\left(i\right)}^{3}+v\left(i\right).$$

**Figure 6:**
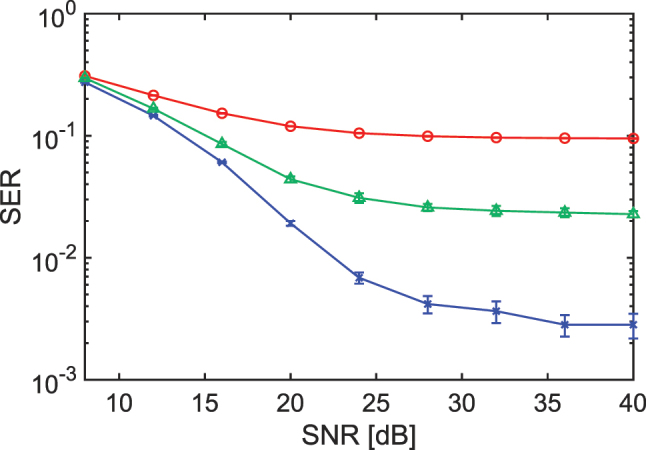
Symbol-error-rate (SER) versus SNR for the NLEQ task. (Red) baseline without the ORC. (Blue) ORC with random inter-mode delays, no added comb-intensity noise. (Green) ORC with both random delays and comb-intensity noise (*σ*_*G*_ = 0.02). Normalized modulation rate and *N*_comb_ are around 4 and 60, respectively. Error bars denote one standard deviation across ten delay patterns.

We use *u*(*i*) as the input signal to the RC. In this task the detuning is swept between 12 and 16, the normalized modulation rate is set to about 4 (which corresponds to 100 MSa/s for a 28 MHz linewidth), and *N*_comb_ = 60. We prepare 8,000 samples, half for training and half for testing, and determine the output weights with ridge regression. Without the ORC the symbol-error rate (SER) shown by the red curve in Figure 6 stays near 0.1 even at high SNR because nonlinear distortion and inter-symbol interference cannot be removed. Using the ORC lowers the SER. When no additional noise is added to the comb-mode intensities the SER falls by more than one order of magnitude at SNR values above 24, as shown by the blue curve in Figure 6. The improvement saturates at high SNR, which suggests that the available nonlinearity in the present ORC is already fully exploited since increasing the modulation rate, i.e. enhancing the memory effect, does not yield further gains. When noise is added to the comb-mode intensities the improvement is smaller, as indicated by the green curve. A LPF cannot be used for this task because the signal is broadly distributed in frequency and the filter would remove both signal and noise.

### 3.2 Experimental demonstration

**Figure 7:**
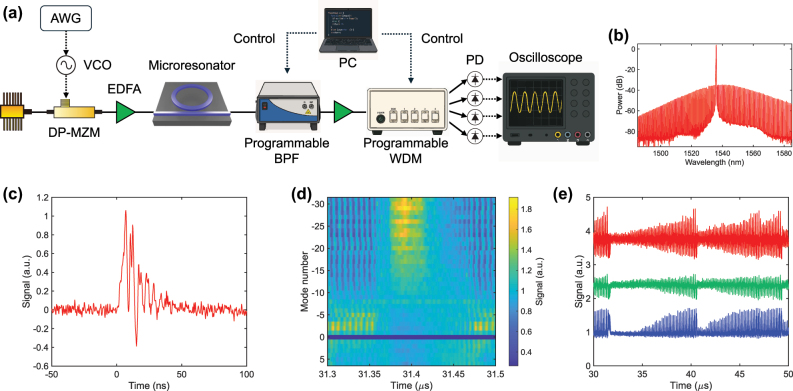
Setup and results of experimental the proposed ORC. (a) Schematic of the experimental setup. AWG, arbitrary waveform generator; DP-MZM, dual-parallel Mach–Zehnder modulator; EDFA, erbium-doped fiber amplifier; BPF, bandpass filter; WDM, wavelength-division multiplexer. (b) Optical spectrum of the soliton comb. RBW is 0.2 nm. (c) AC-coupled photodetected signal of a single comb mode when the detuning is stepped for 5 ns. (d) DC-coupled photodetected signal of the used comb modes when a Santa Fe chaotic signal drives the detuning at 50 MSa/s. The signals are low-pass filtered with the cutoff frequency of 8.5 MHz by post-processing. (e) Traces from (d) extracted at the −2nd (blue), −15th (green), and −29th (red), vertically offset for clarity.

Figure 7(a) shows the proof-of-concept experimental setup. In this demonstration, the output weights are applied electronically instead of with microring arrays. A single-frequency CW laser operating at 1,536 nm provides the pump light. The pump CW laser, with an average power of 200 mW, is coupled into a Si_3_N_4_ microresonator that has a loaded *Q* of 7 × 10^6^ [17]. To reach the soliton state the pump frequency is rapidly swept by a dual-parallel Mach–Zehnder modulator (DP-MZM) driven in carrier-suppressed single-sideband mode [26], [27]. When the input signal is applied, a voltage-controlled oscillator driven by an arbitrary waveform generator (SDG6052X, SIGLENT) adds a modulation to the DP-MZM in addition to the static bias that sets the soliton detuning. The modulation waveform is strictly positive, so the detuning only increases from its initial value. After the soliton comb is generated, a programmable bandpass filter (WaveShaper 1000A, FINISAR) selects four comb modes. These modes are amplified by an erbium-doped fiber amplifier (EDFA) and then split by a programmable wavelength-division multiplexer (WSS-2000, santec) so that each selected mode carries 100 μW of optical power. Each mode is detected by a separate photodiode (PDA05CF2, Thorlabs), and the electrical outputs are sampled by an oscilloscope (DHO4804, RIGOL), yielding four reservoir nodes at once. A computer then retunes the filter and multiplexer to additional sets of modes, ultimately covering 37 modes in total, from +6 to +1 and from −1 to −31. The reservoir outputs are multiplied by the trained weights offline. For stable long-term operation the static detuning is maintained by a feedback loop. One comb mode is monitored, and its power is held constant by adjusting the pump frequency, utilizing the one-to-one relationship between comb-mode power and detuning [28], [29]. The feedback bandwidth is less than 100 Hz, far below the modulation rate, so the loop stabilizes only the static detuning and does not affect the dynamic detuning modulation. Figure 7(b) displays the optical spectrum of the soliton comb. The soliton exists over a detuning range of about 550 MHz, which corresponds to a normalized soliton existence range of about 20 and is 25 % narrower than predicted numerically. Figure 7(c) shows the transient response of a single comb mode when the detuning is increased for 5 ns. The power decays slowly because of the photon lifetime in the resonator and exhibits oscillations with a period of roughly 6 ns, consistent with the numerical results in Figure 3. These oscillations arise from the interplay among the Kerr effect, detuning, and thermal dynamics. The responses of all comb modes are then measured while a Santa Fe chaotic signal drives the detuning at a sampling rate of 50 MSa/s, corresponding to a normalized modulation rate of 1.8. Figure 7(d) presents the resulting waveforms after photodetection. The traces vary gradually with the comb index, and representative examples at the −2nd, −15th, and −29th modes are plotted in Figure 7(e) in blue, green, and red. The distinct responses of different modes supply the high-dimensional and nonlinear mapping required for RC. All traces are normalized to compensate for variations in comb-mode power, and these normalized signals are used in the subsequent benchmark tasks.

**Figure 8:**
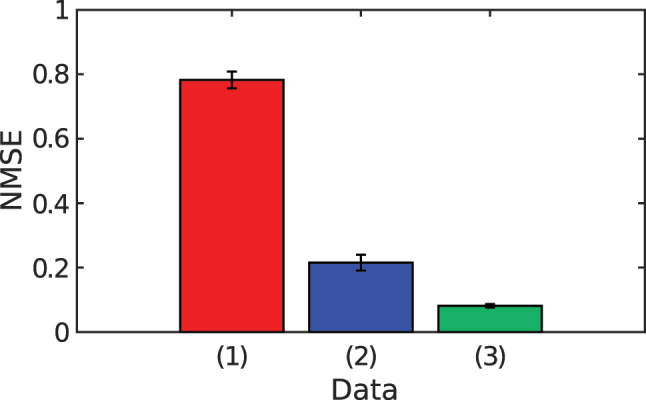
Experimental NMSE for Santa Fe prediction under three post-processing schemes. Red bar (1): no random inter-mode delay, no LPF. Blue bar (2): random delay only. Green bar (3): random delay plus 13.5 MHz LPF. Error bars represent one standard deviation over five independent measurement runs.

The one-step-ahead Santa Fe prediction task is also evaluated experimentally. To obtain reliable statistics, the measurement is repeated five times. As in the numerical study, the NMSE reaches about 0.8 when neither random delay between comb modes nor a LPF is used (trace 1, red, in Figure 8). Introducing a random delay between the comb modes during post-processing lowers the NMSE to 0.21 ± 0.025 (trace 2, blue, in Figure 8). The same delay pattern is applied to all five data sets. Adding a LPF with a 13.5 MHz cut-off further reduces the NMSE to 0.081 ± 0.0056 (trace 3, green). By choosing a more suitable random delay through trial and error we obtain an NMSE as low as 0.061. The experimental errors remain higher than the numerical ones because the measured soliton comb contains more noise than its simulated counterpart. Later we describe how this noise can be reduced to the level assumed in the simulations.

**Figure 9:**
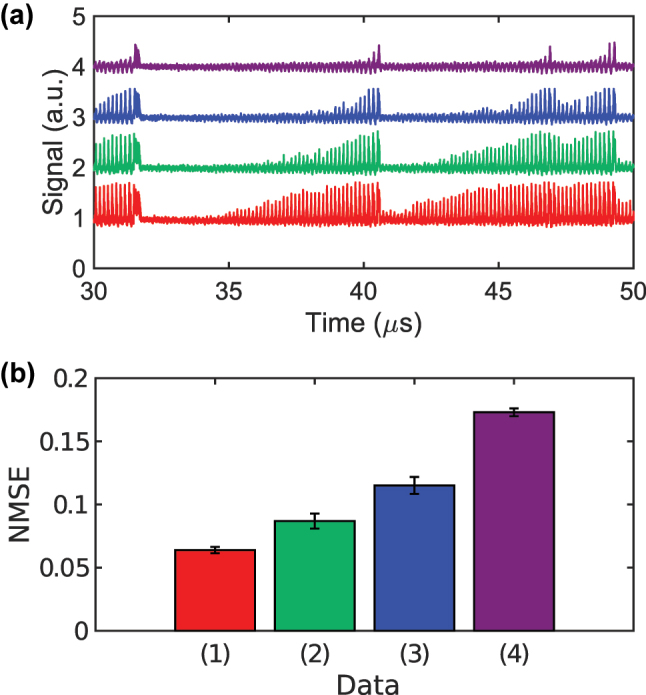
Effect of the initial detuning on reservoir performance. (a) Photodetected signal of the −2nd comb mode for four initial detuning settings. The red trace lies near the blue edge of the soliton existence window; successive shifts toward longer wavelength produce the green, blue and purple traces. (b) Corresponding NMSE for each initial detuning. Colors match (a). Error bars indicate one standard deviation over five data sets. NMSE is evaluated with a fixed random inter-mode delay and a 13.5 MHz LPF.

To minimize NMSE the static detuning must be optimized. Figure 9(a) plots the photodetected signal from the −2nd comb mode while the initial detuning is swept, with the modulation range held constant. From red to green to blue to purple traces the initial setting is shifted toward longer wavelengths, and the response amplitude decreases. This behavior occurs because the comb-mode power is more sensitive to detuning changes at the shorter-wavelength side of the soliton-existence window, as indicated in Figure 2(c). As a result, the NMSE increases, as shown in Figure 9(b). When the initial detuning lies near the blue edge (red data in Figure 9(a) and (b)) the NMSE is 0.064 ± 0.0025. In contrast, when the initial detuning is near the red edge (purple data) the NMSE rises to 0.17 ± 0.0031.

**Figure 10:**
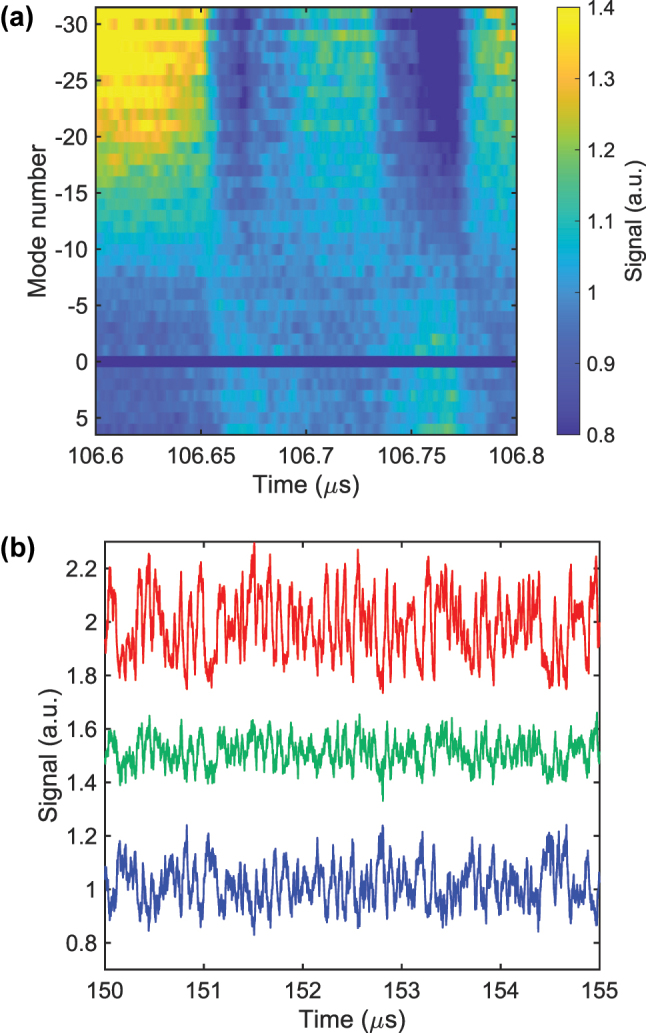
Photodetected comb-mode responses in the NLEQ experiment. (a) Time-domain traces of all measured comb modes when the distorted input defined by Eq. (5) drives the detuning. The modulation sampling rate is 50 MSa/s. (b) Representative modes taken from (a) at the +6th (blue), −12th (green), and −19th (red), vertically offset for clarity.

The nonlinear equalization task is also verified experimentally. The SNR of the distorted input is fixed at 40, and the modulation sampling rate is 50 MSa/s. A total of 4,000 samples are used for training and another 4,000 for testing. Figure 10(a) shows the photodetected traces of every comb mode. The responses vary from mode to mode, confirming that the reservoir maps the one-dimensional input into a high-dimensional space. This contrast is highlighted in Figure 10(b), which plots representative modes at +6 (blue), −12 (green), and −19 (red). In this task, a random delay can be inserted between comb modes, but a LPF cannot be applied because the signal power is widely spread in frequency, so filtering removes useful information. Nonetheless, the measured SER is down to 0.0635 ± 0.0036. The numerical study in Figure 6 indicates that further suppression of intensity noise would lower the SER.

## 4 Discussion and conclusion

Noise currently limits the performance of the demonstrated ORC. Tasks such as NLEQ, where the frequency spectrum of an input signal is broad, are especially sensitive because a LPF cannot be applied. At present the SNR of the reservoir outputs is constrained not by oscilloscope quantization but by ASE from the EDFA that follows the programmable bandpass filter. The measured SNR lies between 20 dB and 25 dB within an RBW of 0.02 nm, corresponding to an input power of about 1 μW per comb mode to the EDFA. Note that although an EDFA is used in the present experiment at telecom wavelengths, any optical amplifier required for combs at other wavelengths would introduce the same noise issue. To rise above the quantization noise of a seven-bit ADC the power per comb mode, *P*_comb_, should reach a few hundred μW. Several measures can raise *P*_comb_. First, an over-coupled resonator increases the out-coupled comb power [30]. Second, a wider comb-mode spacing can be adopted. For a fixed optical bandwidth this choice reduces the total number of modes, but adjacent modes are likely to be less correlated, so fewer modes may still deliver comparable performance. Third, using a coupled-ring resonator with a mode split at the pump enhances the pump-to-comb conversion efficiency [31]. Fourth, dark-soliton combs [32], [33], another mode-locked state of microcombs, could replace bright solitons. Because dark solitons exhibit higher pump-to-comb conversion efficiency, they would increase the comb-mode power and hence the SNR available to the reservoir. Fifth, distributed-feedback lasers can be injection-locked to the comb modes to boost their power [34], [35]. Taken together, these techniques should make it possible to achieve comb modes with an SNR above 40 dB at an RBW of 0.02 nm.

Both the numerical and experimental studies rely on a high-*Q* microresonator with a loaded *Q* of 7 × 10^6^ and a 28 MHz linewidth in order to obtain a wide normalized soliton existence range. A high-*Q* cavity, however, limits the allowable modulation sampling rate because the tasks favour short memory. A moderate-*Q* resonator would support faster sampling at the cost of higher pump power. Considering the need for stronger comb-mode power, an over-coupled microresonator with high intrinsic *Q* and moderate loaded *Q* appears to be the best compromise for increasing *P*_comb_ while permitting higher modulation rates.

In both the numerical and experimental demonstrations, the pump power is fixed. However, better performance is expected with a higher pump power, because a larger soliton-existence range allows a wider detuning modulation and therefore a stronger comb-mode response to the input signal.

Performance can be improved by arranging the ORC in parallel or deep configurations [36]. In a parallel layout, several microrings on the same chip each generate a soliton comb while receiving the same input modulation on their pump lasers. Diversity among the resonators can be engineered through differences in free spectral range, integrated dispersion, or resonance linewidth. If the pump frequency does not need to vary widely, a single pump laser can be split spatially to drive all resonators. The comb modes from every soliton source serve as output nodes, and their weights are obtained with ridge regression, so the total node count grows with the number of parallel combs. In a deep configuration, the weighted sum of the output from a first soliton comb becomes the input to a second comb, and the process can be repeated. Each added layer introduces extra nonlinearity and can reduce the prediction error achieved by the previous layer. A hybrid architecture that combines parallel and deep structures can exploit the strengths of both approaches and reach even higher accuracy on many tasks.

Compared with the frequency-multiplexed ORC that relies on an EO comb and a fiber loop [14], our experiment achieves a similar NMSE on the Santa Fe prediction task but exhibits a higher SER on the NLEQ task. Numerical simulations indicate that raising the SNR of the comb modes would allow the proposed ORC to outperform the EO-comb fiber-loop architecture. In the previous frequency-multiplexed ORC, the modulation rate is limited by the fiber loop at tens of MSa/s. By contrast, our ORC can operate an order of magnitude faster, reaching GSa/s when a microresonator with a lower loaded *Q* is used. Moreover, the proposed ORC is well suited for wafer-scale manufacturing and can be realized in a compact, chip-scale form because of its potential for full integration with CMOS-compatible technology.

In summary we have presented and verified a new architecture for frequency-multiplexed ORC. The modes of a soliton microcomb act as reservoir nodes, supplying memory and nonlinearity through the comb-generation dynamics of a nonlinear microresonator. Numerical proof-of-concept studies demonstrate strong performance, reaching an NMSE of 0.015 on the Santa Fe task and reducing the SER by more than 10 dB on the NLEQ benchmark. Numerical and experimental work further show that noise from EDFAs limits accuracy, and we have mitigated this effect with random inter-node delays and low-pass filtering. Because the design is compatible with silicon photonics, we envisage high-performance chip-scale ORC devices produced with standard CMOS-compatible processes.
